# Social stressors and social resources at work and their association with self-reported health complaints among ready-made garment workers in Bangladesh: a cross-sectional study

**DOI:** 10.1186/s12889-022-14173-x

**Published:** 2022-09-22

**Authors:** Annegret Dreher, Rita Yusuf, Hasan Ashraf, Syed A. K. Shifat Ahmed, Christian Strümpell, Adrian Loerbroks

**Affiliations:** 1 Institute of Occupational, Social, and Environmental Medicine, Centre for Health and Society, Faculty of Medicine, University of Düsseldorf, Moorenstraße 5, 40225 Düsseldorf, Germany; 2 International Center for Biotechnology and Health (ICBH), Center for Health Population and Development (CHPD), Independent University, Plot #16, Block B, Aftabuddin Ahmed Road, Bashundhara R/A, Dhaka, 1229 Bangladesh; 3 Department of Anthropology, Jahangirnagar University, Savar, Dhaka, 1342 Bangladesh; 4 Institute of Social and Cultural Anthropology, University of Hamburg, Edmund-Siemers-Allee 1, West, 20146 Hamburg, Germany

**Keywords:** Bangladesh, Cross-sectional study, Working conditions, Ready-made garment workers, Poisson regression, Employee health, Self-reported health

## Abstract

**Background:**

Bangladesh is one of the world’s largest garment exporters. Physical working conditions of garment workers are precarious and known to largely affect their health. Research on garment workers’ psychosocial working conditions, however, is scarce. We aimed to quantify psychosocial working conditions of garment workers and possible associations with workers’ health.

**Methods:**

We conducted a cross-sectional survey among 1,118 ready-made garment (RMG) workers in labor colonies in Dhaka, Bangladesh, in February 2021. Descriptive analyses were performed to characterize social stressors (e.g., being bullied at work, poor leadership) and social resources at work (e.g., receiving support at work, vertical trust between management and employees, beneficial leadership) and workers’ health (self-reported overall health and 10 specific health complaints). To examine links of social stressors and social resources with self-reported health outcomes we ran multivariable Poisson regression models yielding prevalence ratios (PR) and 95% confidence intervals (CI).

**Results:**

We found low to moderate levels of workplace bullying and high levels of poor leadership (i.e., supervisors not caring about workers’ problems). We also found high levels of social support, vertical trust and beneficial leadership (i.e., supervisors taking decisions free of bias). Garment workers frequently suffered from health complaints, first and foremost headache (68.3%), cold (55.3%), and back pain (50.7%). Health outcomes were poorer among workers who reported to be bullied at work versus not bullied (e.g., PR 1.55 [95% CI 1.32–1.92] for poor self-reported health when bullied by colleagues) and health was better among those reporting to feel supported versus unsupported (e.g., PR 0.61 [0.52–0.71] for poor self-reported health when supported by supervisor). Perceived vertical trust between workers and management was weakly associated with better health. Leadership behavior did not display a consistent pattern.

**Conclusions:**

Our findings suggest that working conditions of RMG workers are rather good (e.g., characterized by low levels of bullying and high levels of support, vertical trust and beneficial leadership). The majority of workers reported good or very good health, although health complaints were frequently mentioned, first and foremost headache, cold, and back pain. Associations between psychosocial working conditions and health indicate worse working conditions being associated with poorer health.

**Supplementary Information:**

The online version contains supplementary material available at 10.1186/s12889-022-14173-x.

## Background

Bangladesh is one of the world’s largest garment exporters with an estimated export value of 35.8 billion USD in 2021 [1]. Over four million mostly female and mostly young workers are employed in Bangladesh’s ready-made garment (RMG) industry [2]. Working conditions of staff in the RMG sector have been described as precarious due to, for instance, low wages [3], long working hours [4], noise exposure [5], unfavorable sitting positions [5], time pressure [6], physical demands [6], and emotional, physical and sexual violence [7–9]. A close link between adverse working conditions and poor employee health is well-established based on extensive evidence amongst various occupational groups across the globe [10–15].

Accordingly, several international studies, including many from Bangladesh, have investigated garment workers’ health and found high prevalences of numerous health complaints, such as musculoskeletal symptoms [6, 16, 17], hearing impairment [18, 19], headache [6, 20], cold [6], sleeplessness [6], and respiratory illness symptoms [21]. Other studies observed depressive symptoms [22], post-traumatic stress disorder [23], anxiety, restlessness and even suicidal thoughts among RMG workers [24]. While the association between physical working conditions (e.g., noise exposure) in the RMG industry and workers’ health has been subject to several studies [16, 18, 25], only few have addressed a possible association between psychosocial working conditions (e.g. support in difficult situations, being treated unfairly at work [26, 27]) and garment workers’ health. In a systematic review Gerhardt et al. (2021) describe social interactions at work as stressors when individuals feel devalued (either in terms of how one is evaluated by others or one’s self-evaluation) [28]. Such stressors were shown to moderately correlate with poorer health-related outcomes among employees [28]. However, studies among different occupational groups have also highlighted that social factors can operate as resources for one’s health. For example, adequate coworker support and a positive relationship with workplace managers have been found to be associated with better employee health [29–31]. With regard to the RMG sector, ethnographic studies among garment workers have likewise highlighted the important role of social factors at work and that these factors may act as both stressors [32, 33] and psychosocial resources [34]. A previous study among Bangladeshi garment workers by our group investigated social support, recognition, and trust in the management at work and found a low level of these resources to be significantly associated with increased odds of several health complaints [6]. This previous study, however, was conducted in one single factory, during working hours of workers and built on a small sample size, which implies potential selection bias and limited statistical power. Moreover, social stressors and resources were not inquired after comprehensively (e.g., no differentiation between social support from colleagues and supervisors, no inquiry after workplace bullying, no inquiry after leadership behavior).

Consequently, both social stressors and social resources can be seen as possible target points of workplace interventions to improve not only working conditions of garment workers, but possibly also their health.

### Aim

To date, no comprehensive epidemiological study of garment workers’ social stressors and social resources at work and their link with health outcomes has been performed to our knowledge. We set out to address this knowledge gap. The aims of this study were, consequently, firstly, to investigate the prevalence of social stressors and social resources at work of RMG workers in Bangladesh. Secondly, we aimed to examine the prevalences of health issues (i.e., poor self-reported health and specific health complaints) among RMG workers and, thirdly, we aimed to investigate possible associations of social stressors and social resources at work with self-reported health outcomes.

## Methods

### Study sample and procedure

Data for this cross-sectional study were collected in four labor colonies located in the area Mirpur, Dhaka, Bangladesh. This area is characterized by a high level of adjacent garment-producing factories and is among the oldest garment factory hubs in Dhaka which is why we thus assumed that many RMG workers live there. Other possible study sites such as Mohammadpur or Jatrabari were rejected as adjacent garment factories were supposed to be less diverse and labor colonies were fewer and more scattered which would have made data collection more difficult. Labor colonies are typically separate slum-like residential areas with unplanned road networks that are densely populated with varying working class inhabitants. Workers often prefer to stay in slums close to their workplace so they can walk to work and save money. The boundaries of the colonies chosen in this study were demarcated through discussions with local residents and community leaders. Based on local newspaper reports and on local informants the total number of garment workers living in the selected colonies was about 6900. Data collection took place between February 10th and March 18th, 2021 by seven female interviewers of a professional survey company. Interviews were performed as fully structured face-to-face interviews with respondents who labeled themselves as garment workers when approached. A computer-assisted personal interviewing (CAPI) method was used and interviews were conducted in the evenings and thus likely after garment workers’ working hours or on weekly holidays. Written informed consent was obtained from all workers. Potential participants were randomly sampled as follows: Interviewers first estimated the total number of households and garment workers of a labor colony with the support of local informants, then divided the number of households by the number of workers which yielded the interval in which households were approached (e.g., 3). The survey team then, starting from one randomly determined corner of the colony, approached household #1 and then subsequently moved on to household #4, then #7, and so on. Similar sampling approaches have been applied in other studies conducted, e.g., in slum settings [35, 36]. In case more than one potential participant was met in a given household, one person was selected at random and invited for the interview. If that person declined, a second person from the household was approached for an interview and so on. As the study was conducted during the SARS-CoV-2 pandemic, all interviewers were provided with personal protective equipment, disinfectants and received appropriate training to ensure adherence to infection control protocols. Eligible for participation were self-labeled garment workers aged 18 and above employed by any garment factory and regardless of their exact type of task within the factory.

### Questionnaire

We designed a questionnaire which measured, among others, sociodemographic characteristics (e.g., sex, age, education, marital status), health behavior (e.g., smoking), employment (e.g., employment duration, type of employment), health outcomes (e.g., self-reported overall health and self-reported health complaints), and social stressors and resources at work.

#### Social stressors and social resources

Social stressors were measured by items inquiring whether one has been bullied by colleagues (yes/no) or supervisors (yes/no), whether supervisors do not care about workers’ problems (yes/no), and whether supervisors take decisions free of personal bias (yes/no). Social resources were measured as receiving support from colleagues (yes/no) or supervisors (yes/no), whether workers trust the information that comes from management (yes/no), and whether the management was perceived to trust workers to do their work well (yes/no).

The rationale to use the abovementioned items largely stems from previous research in the RMG sector, including ethnographic research from our study group [7, 24]. When possible, established items were used, i.e., items on trust were taken from the Copenhagen Psychosocial Questionnaire II [37, 38]. The item on supervisors deciding free of personal bias was taken from a validated organizational justice questionnaire [39]. When established items were not available, they were newly developed by our study team and piloted (see below).

#### Health outcomes

Self-reported health was measured using the single item ‘In general, how would you rate your health?’ rated on a 5-point Likert Scale ‘Very good, good, moderate, bad, very bad’. This item has been demonstrated to predict health status [40] and mortality [41, 42], and correlates with physiological health markers [43, 44]. We also collected data on a predefined set of ten health complaints (i.e., back pain, sleeplessness, headache, breathing problems, cold, tuberculosis, jaundice, stomach problems, muscle cramps, eye problems), which were based on our prior ethnographic [7] and epidemiological research in the garment sector [6]. Participants were asked whether they had been suffering from these complaints in the last two months (yes/no).

#### Translation and piloting of the final instrument

All questionnaire items were discussed and refined within the interdisciplinary author group that combines extensive expertise in the fields of epidemiology and questionnaire development (AL, AD, RY, SA), in ethnographic research with garment workers (CS and HA), and in conducting epidemiological research in Bangladesh (RY and SA). The combination of these different expertises likely increased the face validity and comprehensibility of the questionnaire. The derived set of questions was then translated from English into Bangla by experts of a professional survey company. The questionnaire was then back-translated into English by a bilingual assistant professor. Inconsistencies between translations were discussed by the study team until consensus was reached. The study questionnaire was piloted in a sample of *n* = 56 garment workers from two factories in Dhaka, Bangladesh, to examine whether the length was acceptable and to test how items were understood. The questionnaire was shortened from 111 to 77 items after piloting as it was deemed too long. Furthermore, the wording of some items was adjusted for better comprehensibility and the original 4-point response format to express agreement (Agree a little/ Agree very much/ Disagree a little/ Disagree very much) used for many items was shortened to a binary response format (yes/no) for reasons of time, but also to improve understanding.

### Statistical analysis

Only workers who reported to mainly have worked in the garment sector in the last three months were included in statistical analyses. This criterion was applied post-hoc in addition to the initial inclusion criteria (see above) to ensure that there was a reasonable period of prior exposure to working conditions in a RMG factory. Descriptive analysis of sociodemographic characteristics, health characteristics, social stressors and social resources of the study population was done by displaying absolute numbers and percentages for categorical variables and means and standard deviations (SDs) for continuous variables. Social stressors and social resources at work were grouped into four factors. The construction of these groups largely built on results from tetrachoric correlation analysis performed for the respective dichotomous variables. The four factors were: i) social conflict (variables “You feel bullied by your colleagues on the same rank.” and “You feel bullied by your supervisor.”), ii) leadership (variables “Your supervisors do not care about your problems.” and “Your supervisors take decisions that are free of personal bias.”), iii) social support (variables “Whenever needed you receive support from the colleagues on the same rank” and “Whenever needed you receive support from your supervisor”, and iv) hierarchical interactions at work (variables “You trust the information that comes from the management.” and “The management trusts the employees to do their work well.”).

Robust Poisson regression models were run [45] to identify any association between social stressors and social resources at work with health outcomes. Each variable reflecting social conflict (2 variables), leadership (2 variables), social support (2 variables), and hierarchical interactions at work (2 variables) was analyzed in a separate model. Additionally, a sum score was calculated for each factor to investigate a potential dose–response relationship. Score values were either 0 (indicating e.g., bullying/support from neither colleagues nor supervisor), 1 (e.g., bullying/support from either colleagues or supervisor) or 2 (e.g., bullying/support from both colleagues and supervisor). This led to a final set of 12 independent variables. In line with our prior work among garment workers [6], two types of binary health outcomes served as outcome variables: 1) self-rated overall health and 2) predefined health complaints. The original 5-point scale of self-reported health was dichotomized into “very bad”/ “bad”/ “moderate” (assumed to reflect poor health) health versus “good”/ “very good” health in line with prior work [6]. As mentioned above, we measured a set of ten health complaints. Complaints with a prevalence below 10% were excluded from association analysis (i.e. breathing problems, tuberculosis) as they were deemed not meaningful enough to garment workers and as they may lead to instable statistical models. We therefore analyzed data on eight complaints, i.e. back pain, sleeplessness, headache, cold, jaundice, stomach problems, muscle cramp, eye problems. Two-step adjustment of models was performed, that is, a first model adjusting for age (continuous in years) and sex (male/female) and a second model adjusting for age (continuous in years), sex (male/female), education (no formal education/grade 1–5/grade 6 or higher), marital status (never married/married/separated or divorced or widowed), and smoking status (yes/no). Results of robust Poisson regression analysis are presented as prevalence ratios (PR) with respective 95% confidence intervals (CI). All statistical analyses were done using IBM SPSS Statistics 25.

The exposure to and experience of psychosocial working conditions in the RMG sector may strongly differ by sex and age as suggested by e.g. ethnographic research [46]. We therefore decided to run additional analyses stratified for sex and for age. We grouped the sample in younger and older workers based on the median of the age distribution. We tested for potential interactions by re-running the original Poisson regression models while including and additional interaction term in the respective model (social exposures*sex and social exposures*age, respectively). The *p*-value of the interaction terms was interpreted as indicator of the statistical significance of interactions (significance level of α = 0.05). The results of this additional analysis are presented as additional files (supplementary file 1).

## Results

In total 4,375 households were visited, with at least one garment worker living in 1827 of them. Overall, 1264 garment workers of legal age completed the interview and 1118 of these stated to have mainly worked in the garment sector in the last three months and were included in the analysis. Sociodemographic characteristics of study participants are displayed in Table 1. The study sample was predominantly female (71.3%), young (mean age = 26.2 years, SD = 7.2 years) and had low educational levels (64.1% completed no education or up to grade 5). Prevalences of social stressors and social resources at work are displayed in Table 2. We observed moderate levels of workplace bullying (14.6% and 12.3% reported workplace bullying by colleagues and supervisors, respectively), high levels of poor leadership (44.7% agreed that supervisors did not care about workers’ problems) but also high levels of good leadership (75.7% agreed supervisors took decisions free of personal bias), high levels of social support at the workplace (80.5% reported support from colleagues, 86.0% reported support from supervisors), and high levels of vertical trust between management and employees (82.4% of workers trusted information from the management, 94.1% perceived the management to trust the employees to do their work well). Female and male participants reported similar levels of workplace support and bullying, whereas females reported higher levels of vertical trust and more frequently reported supervisors to be free of personal bias. Younger and older workers reported similar values to all observed social stressors and resources.

**Table 1:** Sociodemographic characteristics of *n* = 1118 ready-made garment workers

**Characteristics**	**Total** **n (%)**	**Women only** **n (%)**	**Men only** **n (%)**	**Younger group**^a^ **n (%)**	**Older group**^a^ **n (%)**
**Sex**					
Female	797 (71.3)	797 (100)	-	433 (71.0)	364 (71.7)
Male	321 (28.7)	-	321 (100)	177 (29.0)	144 (28.3)
**Age, mean (SD)**	26.2 (7.2)	26.3 (7.4)	26.1 (6.9)	21.0 (2.6)	32.5 (6.0)
**Highest level of education**					
No formal education	190 (17.0)	155 (19.4)	35 (10.9)	58 (9.5)	132 (26.0)
Grade 1–5	527 (47.1)	384 (48.2)	143 (44.5)	292 (47.9)	235 (46.3)
Grade 6–10	330 (29.5)	219 (27.5)	111 (34.6)	213 (34.9)	117 (23.0)
Lower secondary exam (matric/SSC)	47 (4.2)	28 (3.5)	19 (5.9)	33 (5.4)	14 (2.8)
Higher secondary exam (Intermediate/HSC)	20 (1.8)	8 (1.0)	12 (3.7)	14 (2.3)	6 (1.2)
Bachelor’s degree	3 (0.3)	2 (0.3)	1 (0.3)	0 (0.0)	3 (0.6)
Postgraduate degree	1 (0.1)	1 (0.1)	0 (0.0)	0 (0.0)	1 (0.2)
**Marital status**					
Married	738 (66.0)	524 (65.7)	214 (66.7)	313 (51.3)	425 (83.7)
Separated or divorced	64 (5.7)	60 (7.5)	4 (1.2)	27 (4.4)	37 (7.3)
Never married	281 (25.1)	178 (22.3)	103 (32.1)	267 (43.8)	14 (2.8)
Husband/wife died	35 (3.1)	35 (4.4)	0 (0.0)	3 (0.5)	32 (6.3)
**Currently smoking tobacco products**					
Yes	279 (25.0)	146 (18.3)	133 (41.4)	94 (15.4)	185 (36.4)
No	839 (75.0)	651 (81.7)	188 (58.6)	516 (84.6)	323 (63.6)

^a^According to age median split at < 26/ ≥ 26 years

**Table 2:** Prevalences of social stressors and social resources at work among *n* = 1118 ready-made garment workers

	**Total**		**Women only**		**Men only**		**Younger group**^b^		**Older group**^b^	
	**n**	**%** **(95% CI**^**a**^**)**	**n**	**%** **(95% CI)**	**n**	**%** **(95% CI)**	**n**	**%** **(95% CI)**	**n**	**%** **(95% CI)**
**Social conflict at work**										
Bullied by your colleagues on the same rank	163	14.6 (12.6- 16.8)	116	14.6 (12.2–17.2)	47	14.6 (11.0–19.0)	80	13.1 (10.5–16.1)	83	16.3 (13.2–19.8)
Bullied by your supervisor	137	12.3 (10.4–14.3)	94	11.8 (9.6–14.2)	43	13.4 (9.9–17.6)	68	11.1 (8.8–13.9)	69	13.6 (10.7–16.9)
**Combined bullying variable**										
Bullied neither by supervisor nor by colleagues	912	81.6 (79.3–83.9)	653	81.9 (79.1–84.5)	259	80.7 (75.9–84.9)	506	83.0 (79.7–85.9)	406	79.9 (76.2–83.3)
Bullied by either supervisor or colleagues	108	9.7 (8.0–11.6)	76	9.5 (7.6–11.8)	32	10.0 (6.9–13.8)	58	9.5 (7.3–12.1)	50	9.8 (7.4–12.8)
Bullied by both, supervisor, and colleagues	96	8.6 (7.0–10.4)	67	8.4 (6.6–10.6)	29	9.0 (6.1–12.7)	45	7.4 (5.4–9.7)	51	10.0 (7.6–13.0)
**Leadership at work**										
Supervisors do not care about workers' problems	500	44.7 (41.8–47.7)	343	43.0 (39.6–46.6)	157	48.9 (43.3–54.5)	283	46.4 (42.2–50.4)	217	42.7 (38.4–47.1)
Supervisors take decisions that are free of personal bias	846	75.7 (73.0–78.2)	593	74.4 (71.2–77.4)	253	78.8 (73.9–83.2)	473	77.5 (74.0–80.8)	373	73.4 (69.4–77.2)
**Combined leadership variable**										
Neither do supervisors care about problems nor do they take decisions free of bias	115	10.3 (8.6–12.3)	82	10.3 (8.3–12.6)	33	10.3 (7.2–14.1)	54	8.9 (6.7–11.4)	61	12.0 (9.3–15.2)
Supervisors do care about problems or take decisions free of bias	536	48.2 (45.2–51.2)	378	47.4 (43.9–51.0)	158	49.2 (43.6–54.8)	311	51.0 (46.9–55.0)	225	44.3 (39.9–48.7)
Supervisors do care about problems and take decisions free of bias	461	41.5 (38.5–44.4)	332	41.7 (38.2–45.2)	129	40.2 (34.8–45.8)	244	40.0 (36.1–44.0)	217	42.7 (38.4–47.1)
**Social support at work**										
Support from your colleagues on the same rank	900	80.5 (78.1–82.8)	635	79.7 (76.7–82.4)	265	82.6 (78.0–86.5)	508	83.3 (80.1–86.2)	392	77.2 (73.3–80.7)
Support from your supervisor	961	86.0 (83.8–87.9)	686	86.1 (83.5–88.4)	275	85.7 (81.4–89.3)	543	89.0 (86.3–91.4)	418	82.3 (78.7–85.5)
**Combined support variable**										
Supported neither by supervisor nor by colleagues	103	9.2 (7.6–11.1)	70	8.8 (6.9–11.0)	33	10.3 (7.2–14.2)	43	7.0 (5.1–9.4)	60	11.8 (9.1–14.9)
Supported by either supervisor or colleagues	167	14.9 (12.9–17.2)	132	16.6 (14.0–19.3)	35	10.9 (7.7–14.8)	83	13.6 (11.0–16.6)	84	16.5 (13.4–20.1)
Supported by both, supervisor, and colleagues	846	75.7 (73.2–78.3)	594	74.5 (71.4–77.5)	252	78.5 (73.6–82.9)	484	79.3 (75.9–82.5)	362	71.3 (67.1–75.2)
**Hierarchical interactions at work**										
Trust in the information that comes from the management	921	82.4 (80.0–84.6)	679	85.2 (82.5–87.6)	242	75.4 (70.3–80.0)	507	83.1 (79.9–86.0)	414	81.5 (77.8–84.8)
Management trusts the employees to do their work well (perceived)	1052	94.1 (92.6–95.4)	761	95.5 (93.8–96.8)	291	90.7 (86.9–93.6)	577	94.6 (92.5–96.2)	475	93.5 (91.0–95.5)
**Combined trust variable**										
Vertical trust in neither of both directions	37	3.3 (2.4–4.6)	21	2.6 (1.6–4.0)	16	5.0 (2.9–8.1)	1	2.8 (1.6–4.4)	20	3.9 (2.4–6.0)
Vertical trust in one direction	177	15.9 (13.8–18.2)	110	13.8 (11.5–16.4)	67	20.9 (16.6–25.7)	97	15.9 (13.1–19.0)	80	15.7 (12.7–19.2)
Vertical trust in both directions	897	80.7 (78.3–83.0)	665	83.4 (80.7–45.2)	232	72.3 (67.9–77.1)	493	80.8 (77.5–83.9)	404	79.5 (75.8–83.0)

^a^CI confidence interval

^b^According to age median split at < 26/ ≥ 26 years

Health outcomes are presented in Table 3. Most participants reported their health to be good or very good (62.1%). The most frequently reported health complaints were headache (68.3%), cold (55.3%), and back pain (50.7%). All health complaints were more frequently reported by female participants than by male participants with differences being especially prominent for headache, back pain, and muscle cramps. Likewise, older participants reported poor self-reported health and health complaints more frequently.

**Table 3:** Self-reported health outcomes of *n* = 1118 ready-made garment workers

Health outcomes	Total		Women only		Men only		Younger group^b^		Older group^b^	
	**n**	**%** **(95%CI**^**a**^**)**	**n**	**%** **(95%CI)**	**n**	**%** **(95%CI)**	**n**	**%** **(95%CI)**	**n**	**%** **(95%CI)**
**Self-reported health**										
Very good	198	17.7 (15.5–20.1)	130	16.3 (13.8–19.1)	68	21.2 (16.8–26.1)	125	20.5 (17.4–23.9)	73	14.4 (11.4–17.7)
Good	496	44.4 (41.4–47.3)	337	42.3 (38.8–45.8)	159	49.5 (43.9–55.1)	291	47.7 (43.7–51.8)	205	40.4 (36.1–44.8)
Moderate	331	29.6 (26.9–32.4)	251	31.5 (28.3–34.8)	80	24.9 (20.3–30.0)	156	25.6 (22.2–29.2)	175	34.3 (30.3–38.8)
Bad	72	6.4 (5.1–8.0)	61	7.7 (5.9–9.7)	11	3.4 (1.7–6.0)	31	5.1 (3.5–7.1)	41	8.1 (5.9–10.8)
Very bad	21	1.9 (1.2–2.9)	18	2.3 (1.3–3.5)	3	0.9 (0.2–2.7)	7	1.1 (0.5–2.4)	14	2.8 (1.5–4.6)
**Prevalence of self-reported health complaints in the past 2 months**										
Back pain	567	50.8 (47.7–53.7)	433	54.3 (50.8–57.8)	134	41.7 (36.3–47.4)	292	47.9 (43.8–51.9)	275	54.1 (49.7–58.5)
Sleeplessness	343	30.7 (28.0–33.5)	246	30.9 (27.7–34.2)	97	30.2 (25.2–35.6)	153	25.1 (21.7–28.7)	190	37.4 (33.2–41.8)
Headache	764	68.3 (65.5–71.1)	571	71.6 (68.4–74.8)	193	60.1 (54.5–65.5)	413	67.6 (63.8–71.4)	351	69.1 (64.9–73.1)
Breathing problems	74	6.6 (5.2–8.2)	65	8.2 (6.4–10.3)	9	2.8 (1.3–5.3)	32	5.2 (3.6–7.3)	42	8.3 (6.0–11.0)
Cold	618	55.3 (52.3–58.2)	457	57.3 (53.8–60.8)	161	50.2 (44.6–55.8)	324	53.1 (49.1–57.1)	294	57.9 (53.4–62.2)
Tuberculosis	9	0.8 (0.4–1.5)	7	0.9 (0.4–1.8)	2	0.6 (0.1–2.2)	3	0.5 (0.1–1.4)	6	1.2 (0.04–0.26)
Jaundice	497	44.5 (41.5–47.4)	363	45.5 (42.0–49.1)	134	41.7 (36.3–47.4)	259	42.5 (38.5–46.5)	238	46.9 (42.4–51.3)
Stomach problems	323	28.9 (26.2–31.6)	244	30.6 (27.4–33.9)	79	24.6 (20.0–29.7)	160	26.2 (22.8–29.9)	163	32.1 (28.0–36.3)
Muscle cramps	477	42.7 (39.7–45.6)	363	45.5 (42.0–49.1)	114	35.5 (30.3–41.0)	234	38.4 (34.5–42.4)	243	47.8 (43.4–52.3)
Eye problems	253	22.6 (20.2–25.2)	194)	24.3 (21.4–27.5)	59	18.4 (14.3–23.1)	105	17.2 (14.3–20.4)	148	29.1 (25.2–33.3)

^a^CI confidence interval

^b^According to age median split at < 26/ ≥ 26 years

Estimates of the associations of social stressors and resources with health outcomes can be found in Table 4. Bullying by either colleagues or supervisors were both significantly associated with poor self-reported health (PR = 1.55 [95% CI = 1.32–1.92] and PR = 1.48 [95% CI = 1.23–1.77], respectively). The associations between bullying and all other investigated health complaints were moderate. Leadership behavior of supervisors displayed an inconsistent pattern. We found a positive association between supervisors not caring about workers’ problems and poor self-reported health (PR = 1.12 [95%CI = 0.96–1.30]), but a significant negative association with supervisors taking decisions free of personal bias (PR = 0.76 [95%CI = 0.65–0.89]) and poor self-reported health. Likewise, inconsistent patterns were observed for the other health outcomes (PRs ranging from 0.80–1.15). Regarding social resources, receiving support by colleagues or supervisors at work were both significantly negatively associated with poor self-reported health (PR = 0.76 [95% CI = 0.65–0.90] and PR = 0.61 [95% CI = 0.52–0.71], respectively). Associations for the other health complaints showed a general pattern of moderate to weak associations (PRs ranging from 0.61–0.99). Vertical trust was also associated with better self-reported health, but only significantly for employees’ trust in the management (PR = 0.74 [95% CI = 0.62–0.87]). Regarding all other health complaints, we observed only weak associations (PRs ranging from 0.75–1.00). With respect to the investigated sum scores of independent variables, the study data did not suggest consistent dose–response relationship in three of the four investigated groups (support, leadership, vertical trust). With respect to bullying, PRs were slightly higher in the category ‘bullied by both colleagues and supervisor’ compared to ‘bullied by either colleagues or supervisor’.

**Table 4:** Association between social stressors and resources with self-reported health complaints among *n* = 1118 ready-made garment workers

	Social conflict at work				Leadership at work				
	Bullied by colleagues	Bullied by supervisor	Bullied by either colleagues or supervisor (vs. neither nor)	Bullied by both colleagues and supervisor (vs. neither nor)	Supervisors do not care about workers’ problems	Supervisors take decisions free of personal bias	Supervisors do care about problems or take decisions free of bias (vs. neither nor)		Supervisors do care about problems and take decisions free of bias (vs. neither nor)
	PR (95% CI)^a^	PR (95% CI)	PR (95% CI)	PR (95% CI)	PR (95% CI)	PR (95% CI)	PR (95% CI)		PR (95% CI)
Poor SRH	**1.55 (1.32–1.92)**	**1.48 (1.23–1.77)**	1.21 (0.96–1.53)	**1.72 (1.43–2.07)**	1.12 (0.96–1.30)	**0.76 (0.65–0.89)**	**0.74 (0.61–0.91)**		**0.66 (0.53–0.82)**
Back pain	**1.22 (1.06–1.40)**	**1.34 (1.16–1.54)**	**1.27 (1.07–1.49)**	**1.30 (1.10–1.55)**	1.08 (0.96–1.21)	0.96 (0.84–1.10)	**0.79 (0.67–0.93)**		**0.82 (0.69–0.97)**
Sleeplessness	**1.62 (1.33–1.97)**	**1.43 (1.14–1.79)**	**1.39 (1.08–1.79)**	**1.66 (1.30–2.12)**	1.09 (0.92–1.31)	**0.69 (0.58–0.83)**	**0.69 (0.54–0.87)**		**0.61 (0.48–0.79)**
Headache	**1.14 (1.03–1.25)**	**1.15 (1.03–1.27)**	1.07 (0.94–1.21)	**1.19 (1.06–1.33)**	0.97 (0.89–1.05)	0.95 (0.87–1.04)	0.96 (0.84–1.09)		0.98 (0.85–1.12)
Cold	**1.21 (1.06–1.38)**	**1.18 (1.02–1.36)**	1.13 (0.96–1.33)	**1.25 (1.06–1.46)**	**0.87 (0.78–0.97)**	**0.84 (0.75–0.94)**	0.87 (0.74–1.03)		0.93 (0.79–1.09)
Jaundice	1.04 (0.87–1.24)	0.97 (0.79–1.19)	0.95 (0.76–1.19)	1.04 (0.82–1.30)	0.90 (0.78–1.03)	**0.82 (0.71–0.94)**	0.88 (0.71–1.08)		0.90 (0.72–1.11)
Stomach problems	**1.47 (1.19–1.83)**	**1.49 (1.19–1.88)**	1.01 (0.73–1.40)	**1.75 (1.38–2.22)**	0.83 (0.69–1.00)	1.10 (0.88–1.38)	1.29 (0.89–1.88)		1.45 (0.99–2.10)
Muscle cramp	**1.65 (1.43–1.90)**	**1.70 (1.47–1.96)**	**1.52 (1.26–1.82)**	**1.83 (1.56–2.15)**	**0.86 (0.75–0.99)**	**0.80 (0.69–0.93)**	**0.76 (0.62–0.93)**		0.85 (0.70–1.04)
Eye problems	**1.48 (1.15–1.90)**	**1.38 (1.04–1.85)**	1.12 (0.79–1.60)	**1.63 (1.20–2.21)**	1.15 (0.93–1.43)	0.93 (0.73–1.19)	1.07 (0.75–1.51)		0.86 (0.60–1.24)
	Social support at work				Hierarchical interactions at work				
	Support from colleagues	Support from supervisor	Support from either colleagues or supervisor (vs. neither nor)	Support from both colleagues and supervisor (vs. neither nor)	Trust in information from management	Management trusts the employees (perceived)	Vertical trust in one direction (vs. neither nor)	Vertical trust in both directions (vs. neither nor)	
	PR (95% CI)	PR (95% CI)	PR (95% CI)	PR (95% CI)	PR (95% CI)	PR (95% CI)	PR (95% CI)	PR (95% CI)	
Poor SRH	**0.76 (0.65–0.90)**	**0.61 (0.52–0.71)**	**0.74 (0.59–0.94)**	**0.60 (0.50–0.72)**	**0.74 (0.62–0.87)**	0.78 (0.58–1.05)	1.14 (0.75–1.74)	0.80 (0.54–1.20)	
Back pain	**0.86 (0.76–0.98)**	**0.84 (0.73–0.97)**	0.95 (0.78–1.17)	**0.82 (0.69–0.98)**	0.88 (0.76–1.01)	**0.80 (0.64–0.99)**	0.93 (0.69–1.25)	0.80 (0.61–1.06)	
Sleeplessness	**0.75 (0.61–0.91)**	**0.69 (0.56–0.85)**	0.94 (0.70–1.26)	**0.68 (0.53–0.87)**	**0.80 (0.65–0.99)**	1.00 (0.68–1.47)	1.03 (0.64–1.66)	0.85 (0.55–1.33)	
Headache	**0.87 (0.80–0.95)**	**0.88 (0.80–0.97)**	1.00 (0.87–1.15)	0.86 (0.76–0.98)	0.95 (0.86–1.05)	0.93 (0.79–1.10)	0.89 (0.73–1.10)	0.87 (0.73–1.05)	
Cold	**0.85 (0.76–0.96)**	0.95 (0.82–1.10)	1.23 (1.00–1.50)	0.96 (0.80–1.16)	0.98 (0.85–1.13)	**0.82 (0.68–0.99)**	1.01 (0.75–1.36)	0.93 (0.71–1.23)	
Jaundice	**0.84 (0.72–0.98)**	**0.80 (0.68–0.94)**	0.94 (0.75–1.19)	**0.79 (0.65–0.95)**	0.95 (0.80–1.13)	0.81 (0.64–1.04)	0.96 (0.67–1.37)	0.88 (0.63–1.22)	
Stomach problems	0.82 (0.66–1.01)	0.99 (0.76–1.29)	1.35 (0.94–1.94)	0.99 (0.71–1.37)	0.90 (0.71–1.14)	0.77 (0.54–1.09)	0.90 (0.55–1.46)	0.79 (0.51–1.24)	
Muscle cramp	0.86 (0.74–1.01)	**0.74 (0.63–0.87)**	1.10 (0.86–1.39)	0.81 (0.66–1.01)	0.98 (0.81–1.17)	0.96 (0.71–1.30)	0.97 (0.65–1.46)	0.95 (0.65–1.38)	
Eye problems	0.86 (0.67–1.09)	0.79 (0.61–1.02)	**0.66 (0.45–0.98)**	**0.71 (0.54–0.94)**	**0.75 (0.58–0.97)**	0.96 (0.58–1.59)	1.72 (0.81–3.65)	1.17 (0.57–2.43)	

*SRH* Self-reported health

^a^Poisson regression results in form of prevalence ratios (PR) with respective 95% confidence intervals (CI). Adjusted for age, sex, marital status, education and tobacco use. Significant findings highlighted in bold

### Stratified analysis according to sex and age

Stratified analyses according to sex indicated slightly stronger associations of bullying and support at work with health outcomes for men. Vertical trust and leadership, however, did not display consistent sex-specific patterns. Analyses stratified for age (according to a median split at 26 years) showed the same patterns indicating worse health when being bullied for the younger group of workers and better health when being supported. When testing for significance of these differences by adding interaction terms to the regression models, only few of these interaction terms were significant. Significant interactions were found e.g., between bullying and sex for muscle cramps (*p*-value bullying by colleagues*sex 0.00, bullying by supervisors*sex 0.01) and between bullying and age for jaundice (*p*-value bullying by colleagues*age 0.04, bullying by supervisors*age 0.02) and support and age for stomach problems (*p*-value support from colleagues*age 0.05, support from supervisor*age 0.04).

## Discussion

The aims of the present study were to examine, firstly, the prevalence of social stressors and social resources at work of RMG workers in Bangladesh and, secondly, possible associations of social stressors and resources with health outcomes. Our study suggests low to moderate levels of workplace bullying, high levels of beneficial leadership (supervisors taking decisions free of bias) but also high levels of poor leadership (supervisors do not care about workers’ problems), high levels of social support at the workplace, and high levels of vertical trust between management and employees. Garment workers frequently suffered from health complaints, first and foremost headache, cold, and back pain. Association analysis indicated worse health among workers who report being bullied at work. Leadership behavior displayed an inconsistent pattern across different health outcomes. Results suggested better worker health when being supported. Finally, vertical trust was weakly associated with better health, although not constantly significant.

### Prevalences of social stressors and social resources at work

We found low to moderate prevalences of workplace bullying (12.3% and 14.6%) and only few participants reported to be bullied by both supervisors and colleagues. Other studies conducted among RMG workers reported prevalences of e.g. physical harassment at work of 8.6%, mental harassment of 25.2% [47], shouting at workers of 57.5% and workers being pushed or shoved of 12.1% [8]. The differences in these observed prevalences may result from differences in the inquired constructs. As the respective constructs also differ from the assessment of bullying used in this study we cannot compare our numbers more specifically with those from other studies. Possibly, also country-specific differences may play a role as demonstrated in a study among RMG workers that found workplace emotional aggression to differ according to the country under study [48]. An explanation for the low to moderate bullying prevalences may also be that rough interaction in the workplace are daily practice and RMG workers considered this as part of their job. Qualitative studies have illustrated e.g. humiliating treatment by supervisors as part of working life of RMG workers [5, 9, 33]. In our study no significant difference between bullying of female and male workers could be observed, which is in contrast to studies that reported female employees to be bullied more frequently [49, 50].

Furthermore, in this study, about half of the supervisors were reported not to care about workers’ problems. A concept suitable for describing a lack of supervisors’ interest in workers may be laissez-faire leadership [51]. This type of leadership is characterized by, among others, a lack of feedback, rewards, and involvement of leaders [51]. Prevalences of destructive leadership behavior including laissez-faire leadership among other occupational groups have been reported at 33.5 to 61.0% [52] and therefore similar to the observed values in this study. Furthermore, three quarters of supervisors were perceived to take decisions free of personal bias by our study sample. This measure resembles the established concept of procedural justice. According to the Organizational Justice model by Greenberg [53] procedural justice describes whether an organization’s processes of decision-making are deemed fair. Several studies have investigated levels of procedural justice across different occupational groups [54–56]. The high level of approval to assumedly bias-free decision making in our study may be due to the fact that for garment workers more substantial stressors (e.g. physical violence [8], job insecurity [6] or payment irregularities [33]) play a more important role than the judgement of fair organizational procedures.

Regarding social resources at work, our study suggests high levels of support from colleagues and supervisors. Three out of four workers even reported to be supported by both colleagues and supervisors. These findings are in line with our previous study among RMG workers that reports similarly high levels of support at work [6] and other international studies from different occupations [57, 58]. However, literature suggests there are different types of workplace support (i.e., emotional support, esteem support, instrumental support, informational support, and network support) [59]. As we did not inquire for specific sub dimensions of support in our study, it remains unclear to which type of support garment workers in our study referred to when reporting support at work.

We furthermore found high levels of perceived vertical trust between the management and the workers. Our results suggested that workers showed slightly less confidence in their management than they reported their management to have towards them. A previous study by our group among workers in a RMG factory revealed even higher levels of trust [6] though it is possible that trust values in our study were lower as workers were not inquired at their workplace, but at their homes which may have made them speak more freely. Nevertheless, the observed trust levels in this study are much higher compared to the levels reported in studies from other countries (e.g., 67.0% trust in supervisors among Finnish employees [60] and 17.5% high organizational trust among Iranian nurses [61]). The observed trust levels also contrast with ethnographic and qualitative studies that report RMG workers being mistrusted by their management and suspected of stealing [7, 62]. Possibly, social desirability as well as culturally influenced response behavior may have led to high values of agreement. It furthermore remains unclear which type of information workers referred to when stating they trusted this information from the management. Information on work-related consequences (e.g., being dismissed when not performing adequately) may be highly trustable whereas promises (e.g., an increase in salary) may not be trusted by workers.

In summary, our study findings suggest rather good working conditions of RMG workers with low levels of bullying and high levels of support. Our results seem to contradict the widespread reporting of precarious working conditions among RMG workers [5, 7, 9]. Possibly, an increase in international attention and pressure towards the garment sector during the last decade may have played a role. This can be seen by e.g., new inspection regimes on fire and building safety of factories that were signed by European and north American buyers [63]. Furthermore, our study was conducted in early 2021 during the COVID-19 pandemic. At the beginning of the pandemic, garment factories in Bangladesh were closed due to national lockdown and orders of up to 3 billion $ were cancelled by international buyers [64]. In consequence, thousands of RMG workers lost their jobs and took to the streets and demanded payment [65–67]. Factories had reopened at the time of conduction of this study, which may have skewed workers’ attitudes towards their workplaces in the way that they viewed it more positively out of relief of being employed again.

### Prevalences of health complaints

Slightly more than one third of participants (37.9%) reported poor self-rated health. Similar values have been observed in a previous study by our group among Bangladeshi RMG workers (40.7%) [6]. Regarding further health complaints, we found high prevalences, especially for headache, cold, and back pain. These findings are in line with other studies among Bangladeshi garment workers who also reported high prevalences of headache [20] and musculoskeletal pain [17], but in contrast to one study that found RMG workers to report back pain only half as often [16]. The health complaints inquired after in this study have also largely been investigated in the previous study by our group [6]. This previous study found only half as much back pain (26.2% (Steinisch et al. (2013) vs. 50.7% (this study)), slightly less sleeplessness (22.3% vs 30.7%), less headache (48.2% vs 68.3%), similar levels of cold (51.8% vs 55.3%), less stomach problems (16.3% vs 28.9%), less muscle cramps (26.0% vs 42.7%) and much less jaundice (6.0% vs 44.5%). Levels of tuberculosis and eye problems were not inquired for and therefore cannot be compared. Nevertheless, it must be kept in mind that this previous study had been conducted within a garment factory and not in a labor colony setting. Workers inquired in factories may refrain from reporting health symptoms as they may fear their employer to discover study results and treat them differently. Furthermore, a healthy worker effect may bias results as workers may not attend work at factories when sick, but may be encountered at home in colonies. Moreover, in terms of selection bias, it may be assumed that mainly factories with better working conditions take part in research studies. These better working conditions may lead to better worker health. In contrast, workers encountered in labor colonies may also be likely to work in factories with mixed working conditions (including factories with very poor conditions) and thus suffer from poorer health. Regarding the high prevalence of cold symptoms observed in this study it cannot be clarified to what extent these may be attributable to COVID as this was not inquired for. Our results suggest better health among male garment workers than among female workers for both self-reported overall health and for all inquired more specific health complaints. One reason may be the double workload of married female workers (i.e., occupational responsibilities and family responsibilities) consuming women’s health. In our study sample, male workers were paid more (data not shown) which may also positively influence their health. Likewise, male workers may receive better nutrition, better care and preferential treatment by their families. Another reason may be that male interviewees may have been less likely to disclose their health concerns to the female interviewers as it may be seen as weakness.

### Association of social stressors and resources with health

Regarding bullying at work, both, bullying by colleagues and bullying by supervisors were consistently associated with worse health and across all investigated health outcomes. Previous systematic reviews among different occupational groups have also demonstrated an association of workplace bullying with both mental and physical health complaints among employees [53, 68]. Our findings suggest a slight dose–response relationship between workplace bullying and health outcomes which has also been reported in other studies [69, 70]. Differences in PRs were, however, small. Possibly, to garment workers, the fact whether one is bullied at all plays a more important role than by how many perpetrators.

Concerning leadership behavior, no clear pattern was identified regarding possible associations with health outcomes. It may be discussed whether garment workers consider being left alone by their supervisors as beneficial which may explain an increase in certain health complaints with an increase in supervisor involvement. Possibly, garment workers also do not expect their supervisors to get involved with workers’ private issues. Few studies have so far investigated an association between biased supervisor decisions and employee health. Some studies have reported an increase in mental health problems among employees reporting lower procedural justice in their companies [71–73]. However, our results cannot confirm this body of literature.

With respect to social support at work, both support by colleagues and support by supervisors in this study were weakly but consistently associated with better health. This is in accordance with other studies that report an inverse relationship between support at work and poor self-rated employee health [74, 75]. In contrast to other studies that found a dose–response relationship between social support at work and self-rated health [13] we could not observe such in our data. Possibly, supervisors and colleagues provide different forms of support that cannot easily be added up. Likewise, it is possible that one of both sources of supports outweighs the other which is supported by a study that found supervisor support to be strongly associated with job satisfaction, whereas colleague support was not [76].

With regard to vertical trust at work, our findings suggest better health among RMG workers in cases when they reported to trust their management, albeit the overall pattern of association was weak and only partly significant. One study among Danish ambulance personnel and fire fighters found vertical trust at work associated with better self-rated health [31]. A different study found, among others, a feeling of trust among colleagues to be associated with better employee health [30]. Our study adds to this body of literature by suggesting a weak inverse association between vertical trust at the workplace and workers’ health.

Interaction analyses suggested that bullying and support showed more pronounced associations with health among men than women. The same applied to younger garment workers. One may hypothesize that younger workers are under additional pressure due to family issues, e.g., the necessity of earning money for their dowry or to support their young children. Men, in contrast, may be affected more by bullying in a male dominated society as they may not feel able to perform their expected role of the family provider. Being bullied in front of a large number of female colleagues may additionally make them feel a loss of masculinity [46, 63]. In turn, a high degree of support may contribute to men’s feeling of worth and may even suggest the possibility for them to be promoted.

### Strengths and limitations

Several limitations apply to our study. We inquired primarily after physical health complaints (self-reported health can be conceptualized as a summary measure of physical and mental health). However, mental health complaints are also likely associated with workplace stressors and resources [77]. It should also be remembered that physical working conditions are very relevant to health of RMG workers such as body position [25] or noise exposure [18]. We adjusted our analyses for smoking, however, we cannot rule out that there are additional confounders which we did not measure and are unable to consider (e.g., alcohol consumption, dietary intake, lifestyle factors). Self-rated assessments of health status and especially self-reports of specific health complaints may differ from objective measurements and have been shown not always to correlate [71]. Due to the study’s cross-sectional design temporal and potentially causal relationships cannot be determined. Although we achieved a good response rate on the household-level (69.1% of households with garment workers living in them), the exact response rate on individual level remains unclear and may have been lower. Eventually it must be kept in mind that data collection was conducted in the midst of the COVID-19 pandemic. It cannot be excluded that due to the pandemic the working conditions of the workers were atypical and differed from pre-pandemic times. Lastly, there are no nationwide statistics on socio-demographic characteristics of RMG workers in Bangladesh likely because the sector is characterized by frequent informal employment and constant fluctuation. It therefore remains unclear to what extent our study sample is representative of the overall population of RMG workers in Bangladesh.

Nevertheless, our study is characterized by several strengths. We took the best possible steps to increase the representativeness of our study sample. Firstly, due to data collection in labor colonies it was possible to interview workers from several different factories with likely diverse work environments and consequently varying social exposures. Random sampling during data collection reduced the risk of selection bias during participant recruitment and increased the representativeness of the study population compared to the total population of RMG workers. Interviews were conducted in the evenings after working hours or on weekly holidays therefore additionally reducing potential selection bias. As interviews were conducted at workers’ homes in their free time it is likely that answers are to a lesser extent biased by social desirability as compared to interviews conducted at workplaces during work hours. The same applies to a possible healthy worker effect. We interviewed self-labeled garment workers. However, no financial or other form of incentive was offered to participants which makes it unlikely that garment workers have posed as such who are not. Furthermore, the study questionnaire was carefully devised, translated, and piloted before implementation to increase instrument acceptance, length and understanding. Eventually, we were able to interview a considerable number of workers, which provided good statistical power.

## Conclusion

Our findings suggest rather good working conditions of RMG workers with low levels of bullying and high levels of support and vertical trust between management and employees. The majority of workers reported good or very good health, although health complaints were also frequently mentioned, first and foremost headache, cold, and back pain. Our results seem to contradict the widespread reporting of precarious working conditions among RMG workers. Nevertheless, the time point of study conduction and newly adopted safety measures in Bangladesh’s garment industry may have influenced results. Associations between psychosocial working conditions and health show similar patterns as observed in international literature, with worse working conditions being associated with poorer health.

## Supplementary Information

**Additional file 1.**

## Data Availability

The datasets used and/or analyzed during the current study are available from the corresponding author on reasonable request.

## Abbreviations

CAPI: Computer-assisted personal interviewing
CI: Confidence interval
HSC: Higher secondary school certificate
PR: Prevalence ratio
RMG: Ready-made garment
SARS-CoV-2: Severe acute respiratory syndrome coronavirus 2
SD: Standard deviation
SRH: Self-reported health
SSC: Secondary school certificate
USD: US Dollar
