# Supplementary material for: Social stressors and social resources at work and their association with self-reported health complaints among ready-made garment workers in Bangladesh: a cross-sectional study
Source: BMC Public Health. 2022 Sep 22;22:1793. doi: 10.1186/s12889-022-14173-x (PMC9492303; doi:10.1186/s12889-022-14173-x)
Supplement: Supplementary file 1 — Additional file 1. [file 12889_2022_14173_MOESM1_ESM.docx]

**Supplementary Table: Association of social stressors and social resources at work with self-reported health complaints among n= 797 female ready-made garment workers**

|  | Social conflict at work | | | | Social support at work | | | |
| --- | --- | --- | --- | --- | --- | --- | --- | --- |
|  | Bullied by colleagues | Bullied by supervisor | Bullied by either colleagues or supervisor  (sum score) | Bullied by both colleagues and supervisor  (sum score) | Support from colleagues | Support from supervisor | Support from either colleagues or supervisor  (sum score) | Support from both colleagues and supervisor (sum score) |
|  | PR (95% CI)* | PR (95% CI) | PR (95% CI) | PR (95% CI) | PR (95% CI) | PR (95% CI) | PR (95% CI) | PR (95% CI) |
| Poor SRH | **1.46 (1.22-1.76)** | **1.41 (1.16-1.73)** | 1.17 (0.89-1.53) | **1.62 (1.32-1.98)** | **0.82 (0.68-0.98)** | **0.62 (0.52-0.73)** | 0.80 (0.62-1.04) | **0.64 (0.52-0.79)** |
| Back pain | 1.17 (0.99-1.34) | **1.32 (1.12-1.55)** | 1.17 (0.96-1.42) | **1.29 (1.07-1.56)** | 0.92 (0.80-1.07) | **0.85 (0.73-0.99)** | 0.99 (0.79-1.24) | 0.88 (0.72-1.07) |
| Sleeplessness | **1.55 (1.23-1.97)** | **1.38 (1.04-1.82)** | **1.43 (1.06-1.93)** | **1.56 (1.15-2.11)** | 0.80 (0.63-1.01) | 0.78 (0.60-1.01) | 1.12 (0.77-1.61) | 0.80 (0.57-1.11) |
| Headache | 1.07 (0.96-1.20) | 1.09 (0.97-1.23) | 1.02 (0.88-1.19) | 1.12 (0.98-1.28) | 0.91 (0.83-1.01) | 0.92 (0.82-1.03) | 0.97 (0.83-1.14) | 0.89 (0.78-1.02) |
| Cold | 1.17 (1.00-1.36) | **1.23 (1.05-1.44)** | 1.04 (0.85-1.27) | **1.29 (1.08-1.54)** | **0.87 (0.76-0.99)** | 0.95 (0.80-1.12) | 1.16 (0.92-1.47) | 0.95 (0.76-1.18) |
| Jaundice | 1.14 (0.93-1.38) | 0.90 (0.70-1.16) | 1.04 (0.81-1.34) | 1.02 (0.78-1.34) | 0.91 (0.76-1.08) | **0.82 (0.68-0.99)** | 0.97 (0.74-1.29) | 0.84 (0.66-1.07) |
| Stomach problems | **1.37 (1.06-1.76)** | **1.44 (1.10-1.89)** | 0.94 (0.64-1.38) | **1.65 (1.25-2.18)** | 0.83 (0.65-1.06) | 0.94 (0.70-1.27) | 1.34 (0.88-2.03) | 0.98 (0.67-1.44) |
| Muscle cramp | **1.37 (1.15-1.63)** | **1.52 (1.28-1.81)** | 1.25 (0.99-1.57) | **1.57 (1.30-1.90)** | 0.97 (0.80-1.16) | **0.75 (0.62-0.89)** | 0.97 (0.74-1.29) | 0.83 (0.65-1.06) |
| Eye problems | **1.50 (1.14-1.99)** | 1.31 (0.93-1.85) | 1.05 (0.69-1.60) | **1.63 (1.16-2.29)** | 0.81 (0.62-1.06) | 0.83 (0.62-1.12) | **0.49 (0.32-0.77)** | **0.64 (0.48-0.87)** |

*Poisson regression results in form of prevalence ratios (PR) with respective 95% confidence intervals (CI). Adjusted for age, marital status, education and tobacco use. Significant findings highlighted in bold.

**Supplementary Table: Association of social stressors and social resources at work with self-reported health complaints among n= 797 female ready-made garment workers**

|  | Hierarchical interactions at work | | | | Leadership at work | | | |
| --- | --- | --- | --- | --- | --- | --- | --- | --- |
|  | Trust in information from management | Management trusts the employees | Vertical trust in one direction (sum score) | Vertical trust in both directions (sum score) | Supervisors do not care about workers’ problems | Supervisors take decisions free of personal bias | Supervisors do care about problems or take decisions free of bias  (sum score) | Supervisors do care about problems and take decisions free of bias  (sum score) |
|  | PR (95% CI) | PR (95% CI) | PR (95% CI) | PR (95% CI) | PR (95% CI) | PR (95% CI) | PR (95% CI) | PR (95% CI) |
| Poor SRH | **0.79 (0.65-0.96)** | 0.92 (0.62-1.36) | 1.23 (0.69-2.18) | 0.94 (0.54-1.63) | **1.18 (1.01-1.39)** | **0.78 (0.66-0.93)** | **0.75 (0.60-0.94)** | **0.64 (0.51-0.82)** |
| Back pain | 0.96 (0.81-1.14) | 0.82 (0.64-1.06) | 0.82 (0.58-1.17) | 0.80 (0.58-1.10) | 1.09 (0.96-1.24) | 0.91 (0.79-1.05) | **0.82 (0.68-0.98)** | **0.79 (0.66-0.95)** |
| Sleeplessness | 0.89 (0.68-1.12) | 1.21 (0.68-2.16) | 1.44 (0.65-3.17) | 1.25 (0.59-2.67) | 1.10 (0.89-1.35) | **0.69 (0.56-0.85)** | **0.69 (0.52-0.92)** | **0.61 (0.45-0.82)** |
| Headache | 0.95 (0.84-1.06) | 0.90 (0.75-1.06) | 0.85 (0.69-1.06) | 0.83 (0.69-1.01) | 1.01 (0.92-1.10) | 0.97 (0.88-1.07) | 1.03 (0.88-1.20) | 0.99 (0.85-1.16) |
| Cold | 0.96 (0.81-1.13) | **0.69 (0.57-0.82**) | **0.71 (0.54-0.94)** | **0.69 (0.55-0.87)** | **0.86 (0.76-0.98)** | **0.87 (0.77-0.99)** | 0.89 (0.74-1.08) | 0.98 (0.80-1.17) |
| Jaundice | 0.93 (0.76-1.14) | 0.81 (0.59-1.10) | 1.12 (0.68-1.84) | 0.96 (0.60-1.54) | 0.91 (0.78-1.06) | 0.88 (0.74-1.04) | 0.94 (0.73-1.21) | 0.96 (0.74-1.24) |
| Stomach problems | 0.90 (0.68-1.20) | 0.70 (0.46-1.07) | 0.65 (0.38-1.23) | 0.63 (0.39-1.02) | 0.83 (0.67-1.04) | 1.11 (0.86-1.43) | 1.36 (0.88-2.11) | 1.50 (0.97-2.32) |
| Muscle cramp | 1.02 (0.82-1.27) | 0.93 (0.65-1.33) | 0.71 (0.45-1.12) | 0.80 (0.53-1.18) | 0.90 (0.77-1.06) | **0.78 (0.66-0.91)** | **0.73 (0.59-0.91)** | **0.79 (0.64-0.98)** |
| Eye problems | 0.84 (0.61-1.15) | 1.15 (0.57-2.29) | 1.01 (0.44-2.30) | 0.90 (0.41-1.94) | 1.15 (0.90-1.47) | 0.94 (0.71-1.23) | 0.91 (0.63-1.33) | 0.80 (0.54-1.18) |

*Poisson regression results in form of prevalence ratios (PR) with respective 95% confidence intervals (CI). Adjusted for age, marital status, education and tobacco use. Significant findings highlighted in bold.

**Supplementary Table: Association of social stressors and social resources at work with self-reported health complaints among n= 321 male ready-made garment workers**

|  | Social conflict at work | | | | Social support at work | | | |
| --- | --- | --- | --- | --- | --- | --- | --- | --- |
|  | Bullied by colleagues | Bullied by supervisor | Bullied by either colleagues or supervisor  (sum score) | Bullied by both colleagues and supervisor  (sum score) | Support from colleagues | Support from supervisor | Support from either colleagues or supervisor  (sum score) | Support from both colleagues and supervisor (sum score) |
|  | PR (95% CI)* | PR (95% CI) | PR (95% CI) | PR (95% CI) | PR (95% CI) | PR (95% CI) | PR (95% CI) | PR (95% CI) |
| Poor SRH | **1.87 (1.31-2.65)** | **1.58 (1.06-2.35)** | 1.48 (0.91-2.40) | **1.94 (1.23-2.96)** | **0.63 (0.43-0.92)** | **0.64 (0.43-0.96)** | 0.59 (0.31-1.13) | **0.54 (0.36-0.83)** |
| Back pain | **1.40 (1.05-1.88)** | **1.42 (1.05-1.90)** | **1.61 (1.18-2.19)** | 1.37 (0.94-2.00) | **0.67 (0.51-0.89)** | 0.78 (0.56-1.07) | 0.84 (0.53-1.33) | **0.67 (0.48-0.94)** |
| Sleeplessness | **1.83 (1.29-2.60)** | **1.59 (1.09-2.30)** | 1.35 (0.83-2.18) | **1.97 (1.32-2.94)** | **0.62 (0.44-0.87)** | **0.49 (0.34-0.68)** | 0.57 (0.32-1.00) | **0.46 (0.32-0.66)** |
| Headache | **1.34 (1.11-1.61)** | **1.26 (1.03-1.55)** | 1.19 (0.93-1.54) | **1.38 (1.11-1.71)** | **0.76 (0.63-0.92)** | **0.78 (0.63-0.96)** | 1.20 (0.90-1.61) | 0.80 (0.61-1.04) |
| Cold | **1.35 (1.05-1.73)** | 1.06 (0.78-1.44) | **1.39 (1.05-1.84)** | 1.15 (0.80-1.64) | 0.82 (0.64-1.05) | 0.96 (0.70-1.31) | 1.48 (0.98-2.25) | 0.99 (0.68-1.44) |
| Jaundice | 0.99 (0.97-1.01) | 1.15 (0.81-1.62) | 0.73 (0.44-1.22) | 1.08 (0.70-1.65) | **0.70 (0.53-0.91)** | **0.72 (0.53-0.98)** | 0.88 (0.56-1.38) | **0.66 (0.48-0.92)** |
| Stomach problems | **1.89 (1.26-2.85)** | **1.65 (1.06-2.57)** | 1.25 (0.68-2.31) | **2.11 (1.34-3.33)** | 0.77 (0.49-1.22) | 1.05 (0.58-1.89) | 1.27 (0.57-2.81) | 0.93 (0.48-1.79) |
| Muscle cramp | **2.59 (2.01-3.34)** | **2.27 (1.73-2.97)** | **2.57 (1.88-3.52)** | **2.67 (1.98-3.62)** | **0.61 (0.45-0.82)** | 0.74 (0.52-1.06) | **1.65 (1.03-2.64)** | 0.75 (0.48-1.17) |
| Eye problems | 1.45 (0.81-2.61) | 1.56 (0.88-2.79) | 1.38 (0.70-2.72) | 1.63 (0.81-3.28) | 1.10 (0.60-2.03) | 0.67 (0.39-1.15) | 2.12 (0.93-4.85) | 1.10 (0.55-2.21) |

*Poisson regression results in form of prevalence ratios (PR) with respective 95% confidence intervals (CI). Adjusted for age, marital status, education and tobacco use. Significant findings highlighted in bold.

**Supplementary Table: Association of social stressors and social resources at work with self-reported health complaints among n= 321 male ready-made garment workers**

|  | Hierarchical interactions at work | | | | Leadership at work | | | |
| --- | --- | --- | --- | --- | --- | --- | --- | --- |
|  | Trust in information from management | Management trusts the employees | Vertical trust in one direction (sum score) | Vertical trust in both directions (sum score) | Supervisors do not care about workers’ problems | Supervisors take decisions free of personal bias | Supervisors do care about problems or take decisions free of bias  (sum score) | Supervisors do care about problems and take decisions free of bias  (sum score) |
|  | PR (95% CI) | PR (95% CI) | PR (95% CI) | PR (95% CI) | PR (95% CI) | PR (95% CI) | PR (95% CI) | PR (95% CI) |
| Poor SRH | **0.61 (0.43-0.86)** | **0.58 (0.36-0.92)** | 0.96 (0.50-1.84) | 0.56 (0.39-1.04) | 0.90 (0.64-1.27) | **0.67 (0.47-0.97)** | 0.72 (0.43-1.20) | 0.73 (0.44-1.22) |
| Back pain | **0.71 (0.55-0.93)** | 0.74 (0.50-1.09) | 1.17 (0.67-2.04) | 0.77 (0.45-1.33) | 1.01 (0.78-1.31) | 1.16 (0.82-1.65) | 0.70 (0.47-1.04) | 0.90 (0.61-1.33) |
| Sleeplessness | **0.63 (0.45-0.89)** | 0.76 (0.45-1.29) | 0.72 (0.40-1.28) | **0.53 (0.31-0.90)** | 1.04 (0.75-1.45) | **0.68 (0.48-0.97)** | 0.64 (0.41-1.01) | **0.62 (0.39-0.99)** |
| Headache | 0.96 (0.78-1.19) | 1.02 (0.73-1.44) | 0.97 (0.62-1.50) | 0.96 (0.64-1.44) | 0.86 (0.72-1.03) | 0.90 (0.73-1.10) | 0.78 (0.59-1.02) | 0.94 (0.73-1.22) |
| Cold | 1.02 (0.78-1.33) | 1.19 (0.76-1.85) | 2.35 (0.98-5.61) | 1.98 (0.85-4.66) | 0.90 (0.72-1.12) | **0.75 (0.60-0.94)** | 0.82 (0.60-1.12) | 0.84 (0.61-1.15) |
| Jaundice | 0.97 (0.71-1.31) | 0.82 (0.56-1.20) | 0.73 (0.43-1.22) | 0.75 (0.48-1.19) | 0.84 (0.65-1.09) | **0.66 (0.51-0.87)** | 0.73 (0.50-1.08) | 0.77 (0.52-1.14) |
| Stomach problems | 0.85 (0.55-1.32) | 0.92 (0.48-1.77) | 1.76 (0.60-5.15) | 1.26 (0.45-3.55) | 0.78 (0.53-1.16) | 1.04 (0.64-1.69) | 1.12 (0.54-2.34) | 1.35 (0.65-2.80) |
| Muscle cramp | 0.92 (0.66-1.29) | 0.92 (0.53-1.60) | 1.57 (0.63-3.88) | 1.25 (0.52-3.03) | 0.76 (0.56-1.03) | 0.84 (0.60-1.19) | 0.78 (0.47-1.30) | 1.00 (0.61-1.66) |
| Eye problems | **0.57 (0.36-0.92)** | 0.77 (0.37-1.60) | 6.11 (0.92-40.41) | 2.36 (0.36-15.48) | 1.15 (0.72-1.83) | 0.92 (0.55-1.52) | 2.06 (0.81-5.28) | 1.26 (0.47-3.42) |

*Poisson regression results in form of prevalence ratios (PR) with respective 95% confidence intervals (CI). Adjusted for age, marital status, education and tobacco use. Significant findings highlighted in bold.

**Supplementary Table: Association of social stressors and social resources at work with self-reported health complaints among n=610 ready-made garment workers aged 18-25 (younger group according to median split)**

|  | Social conflict at work | | | | Social support at work | | | |
| --- | --- | --- | --- | --- | --- | --- | --- | --- |
|  | Bullied by colleagues | Bullied by supervisor | Bullied by either colleagues or supervisor  (sum score) | Bullied by both colleagues and supervisor  (sum score) | Support from colleagues | Support from supervisor | Support from either colleagues or supervisor  (sum score) | Support from both colleagues and supervisor (sum score) |
|  | PR (95% CI)* | PR (95% CI) | PR (95% CI) | PR (95% CI) | PR (95% CI) | PR (95% CI) | PR (95% CI) | PR (95% CI) |
| Poor SRH | **1.69 (1.30-2.20)** | 1.35 (0.99-1.85) | 1.35 (0.96-1.91) | **1.69 (1.21-2.36)** | **0.70 (0.54-0.92)** | **0.56 (0.43-0.74)** | 0.75 (0.50-1.11) | **0.54 (0.39-0.75)** |
| Back pain | **1.29 (1.05-1.58)** | **1.42 (1.17-1.72)** | **1.34 (1.07-1.69)** | **1.40 (1.09-1.79)** | **0.81 (0.66-0.98)** | **0.73 (0.59-0.90)** | 0.82 (0.60-1.12) | **0.69 (0.53-0.90)** |
| Sleeplessness | **2.00 (1.49-2.69)** | **1.66 (1.17-2.34)** | **1.77 (1.23-2.54)** | **2.01 (1.38-2.95)** | **0.69 (0.50-0.95)** | **0.57 (0.41-0.80)** | 1.12 (0.70-1.81) | **0.63 (0.41-0.97)** |
| Headache | 1.14 (0.99-1.31) | **1.17 (1.02-1.34)** | 1.12 (0.95-1.32) | 1.19 (1.00-1.41) | **0.82 (0.73-0.92)** | **0.78 (0.69-0.88)** | 0.87 (0.73-1.04) | **0.74 (0.64-0.86)** |
| Cold | 1.12 (0.91-1.38) | 1.13 (0.91-1.40) | 1.01 (0.78-1.32) | 1.20 (0.94-1.53) | 0.88 (0.73-1.05) | 0.97 (0.76-1.23) | 1.21 (0.86-1.69) | 0.99 (0.73-1.34) |
| Jaundice | 0.86 (0.63-1.16) | 0.77 (0.54-1.10) | **0.65 (0.43-0.97)** | 0.90 (0.62-1.32) | 0.89 (0.70-1.13) | 1.04 (0.77-1.41) | 1.14 (0.75-1.73) | 1.00 (0.69-1.45) |
| Stomach problems | **1.47 (1.05-2.07)** | **1.46 (1.02-2.08)** | 1.27 (0.84-1.94) | **1.62 (1.07-2.44)** | 0.78 (0.56-1.07) | 0.99 (0.64-1.52) | **1.94 (1.03-3.66)** | 1.18 (0.65-2.15) |
| Muscle cramp | **1.75 (1.41-2.18)** | **1.70 (1.36-2.14)** | **1.63 (1.26-2.11)** | **1.89 (1.44-2.47)** | 0.87 (0.67-1.13) | 0.78 (0.59-1.03) | 1.26 (0.81-1.96) | 0.91 (0.61-1.36) |
| Eye problems | **1.78 (1.16-2.71)** | 1.54 (0.98-2.43) | 1.39 (0.82-2.39) | **1.91 (1.14-3.18)** | 0.99 (0.62-1.58) | 1.04 (0.59-1.84) | 0.98 (0.42-2.27) | 1.00 (0.50-2.01) |

*Poisson regression results in form of prevalence ratios (PR) with respective 95% confidence intervals (CI). Adjusted for sex, marital status, education and tobacco use. Significant findings highlighted in bold.

**Supplementary Table: Association of social stressors and social resources at work with self-reported health complaints among n=610 ready-made garment workers aged 18-25 (younger group according to median split)**

|  | Hierarchical interactions at work | | | | Leadership at work | | | |
| --- | --- | --- | --- | --- | --- | --- | --- | --- |
|  | Trust in information from management | Management trusts the employees | Vertical trust in one direction (sum score) | Vertical trust in both directions (sum score) | Supervisors do not care about workers’ problems | Supervisors take decisions free of personal bias | Supervisors do care about problems or take decisions free of bias  (sum score) | Supervisors do care about problems and take decisions free of bias  (sum score) |
|  | PR (95% CI) | PR (95% CI) | PR (95% CI) | PR (95% CI) | PR (95% CI) | PR (95% CI) | PR (95% CI) | PR (95% CI) |
| Poor SRH | 0.80 (0.60-1.06) | 0.67 (0.45-1.00) | 0.95 (0.51-1.78) | 0.72 (0.40-1.30) | 1.13 (0.89-1.42) | **0.70 (0.55-0.89)** | **0.55 (0.41-0.74)** | **0.53 (0.39-0.73)** |
| Back pain | **0.77 (0.63-0.94)** | **0.60 (0.48-0.75)** | 0.74 (0.53-1.04) | **0.57 (0.42-0.77)** | 1.04 (0.88-1.23) | 0.92 (0.76-1.12) | 0.80 (0.62-1.04) | 0.82 (0.63-1.07) |
| Sleeplessness | 0.91 (0.65-1.29) | 1.01 (0.56-1.84) | 1.44 (0.61-3.39) | 1.22 (0.54-2.76) | 1.12 (0.85-1.47) | **0.59 (0.45-0.78)** | **0.65 (0.45-0.94)** | **0.50 (0.34-0.76)** |
| Headache | 0.88 (0.77-1.00) | 0.87 (0.71-1.06) | 0.95 (0.71-1.26) | 0.83 (0.64-1.09) | 1.00 (0.90-1.12) | 0.91 (0.81-1.02) | 0.87 (0.73-1.03) | 0.87 (0.73-1.04) |
| Cold | 0.91 (0.76-1.10) | 0.82 (0.63-1.07) | 1.00 (0.67-1.50) | 0.88 (0.60-1.29) | 0.87 (0.75-1.01) | 0.89 (0.76-1.05) | 0.90 (0.69-1.17) | 0.99 (0.76-1.28) |
| Jaundice | 0.99 (0.77-1.27) | 0.79 (0.55-1.12) | 0.98 (0.56-1.73) | 0.91 (0.54-1.54) | 0.84 (0.70-1.02) | **0.79 (0.65-0.96)** | 0.80 (0.59-1.08) | 0.88 (0.65-1.19) |
| Stomach problems | 0.77 (0.56-1.07) | 0.77 (0.45-1.32) | 1.56 (0.65-3.75) | 1.05 (0.45-2.47) | 0.87 (0.66-1.13) | 0.93 (0.68-1.27) | 0.96 (0.58-1.57) | 1.05 (0.64-1.73) |
| Muscle cramp | 0.90 (0.69-1.18) | 0.85 (0.55-1.31) | 0.98 (0.53-1.82) | 0.87 (0.49-1.55) | 0.91 (0.74-1.11) | 0.83 (0.67-1.03) | 0.79 (0.58-1.08) | 0.84 (0.61-1.16) |
| Eye problems | **0.60 (0.40-0.88)** | 0.79 (0.38-1.67) | 2.40 (0.63-9.23) | 1.22 (0.32-4.60) | 1.29 (0.91-1.83) | 1.17 (0.76-1.81) | 1.01 (0.55-1.84) | 0.87 (0.46-1.63) |

*Poisson regression results in form of prevalence ratios (PR) with respective 95% confidence intervals (CI). Adjusted for sex, marital status, education and tobacco use. Significant findings highlighted in bold.

**Supplementary Table: Association of social stressors and social resources at work with self-reported health complaints among n=508 ready-made garment workers aged 26 and above (older group according to median split)**

|  | Social conflict at work | | | | Social support at work | | | |
| --- | --- | --- | --- | --- | --- | --- | --- | --- |
|  | Bullied by colleagues | Bullied by supervisor | Bullied by either colleagues or supervisor  (sum score) | Bullied by both colleagues and supervisor  (sum score) | Support from colleagues | Support from supervisor | Support from either colleagues or supervisor  (sum score) | Support from both colleagues and supervisor (sum score) |
|  | PR (95% CI)* | PR (95% CI) | PR (95% CI) | PR (95% CI) | PR (95% CI) | PR (95% CI) | PR (95% CI) | PR (95% CI) |
| Poor SRH | **1.40 (1.14-1.72)** | **1.51 (1.23-1.86)** | 1.11 (0.80-1.54) | **1.63 (1.32-2.02)** | 0.82 (0.67-1.01) | **0.64 (0.53-0.77)** | **0.69 (0.52-0.92)** | **0.63 (0.50-0.78)** |
| Back pain | 1.18 (0.97-1.44) | **1.26 (1.02-1.55)** | 1.20 (0.95-1.52) | 1.24 (0.98-1.58) | 0.90 (0.75-1.07) | 0.91 (0.75-1.09) | 1.00 (0.76-1.31) | 0.89 (0.71-1.12) |
| Sleeplessness | **1.32 (1.01-1.72)** | 1.21 (0.90-1.63) | 1.19 (0.83-1.70) | 1.33 (0.96-1.83) | 0.81 (0.63-1.04) | 0.79 (0.60-1.02) | 0.80 (0.55-1.16) | **0.72 (0.54-0.98)** |
| Headache | 1.14 (1.00-1.31) | 1.12 (0.96-1.30) | 1.00 (0.82-1.23) | **1.19 (1.02-1.39)** | 0.93 (0.82-1.06) | 0.97 (0.84-1.13) | 1.12 (0.91-1.38) | 0.98 (0.81-1.18) |
| Cold | **1.27 (1.08-1.51)** | 1.21 (1.00-1.46) | **1.28 (1.05-1.57)** | **1.26 (1.01-1.56)** | **0.85 (0.72-0.99)** | 0.94 (0.78-1.14) | 1.24 (0.96-1.60) | 0.94 (0.74-1.20) |
| Jaundice | 1.19 (0.96-1.48) | 1.16 (0.91-1.48) | 1.26 (0.97-1.63) | 1.16 (0.88-1.54) | **0.80 (0.66-0.97)** | **0.70 (0.58-0.84)** | 0.86 (0.65-1.13) | **0.69 (0.55-0.86)** |
| Stomach problems | **1.42 (1.07-1.89)** | **1.46 (1.08-1.97)** | 0.77 (0.46-1.28) | **1.74 (1.29-2.34)** | 0.87 (0.65-1.16) | 1.03 (0.74-1.44) | 1.05 (0.67-1.66) | 0.94 (0.63-1.38) |
| Muscle cramp | **1.58 (1.32-1.90)** | **1.65 (1.37-1.98)** | **1.40 (1.07-1.82)** | **1.77 (1.46-2.15)** | 0.85 (0.70-1.04) | **0.72 (0.59-0.87)** | 0.98 (0.73-1.30) | **0.75 (0.59-0.96)** |
| Eye problems | 1.27 (0.92-1.75**)** | 1.18 (0.82-1.71) | 0.94 (0.58-1.52) | 1.36 (0.94-1.99) | 0.79 (0.59-1.06) | **0.69 (0.51-0.94)** | **0.50 (0.31-0.80)** | **0.60 (0.44-0.82)** |

*Poisson regression results in form of prevalence ratios (PR) with respective 95% confidence intervals (CI). Adjusted for sex, marital status, education and tobacco use. Significant findings highlighted in bold.

**Supplementary Table: Association of social stressors and social resources at work with self-reported health complaints among n=508 ready-made garment workers aged 26 and above (older group according to median split)**

|  | Hierarchical interactions at work | | | | Leadership at work | | | |
| --- | --- | --- | --- | --- | --- | --- | --- | --- |
|  | Trust in information from management | Management trusts the employees | Vertical trust in one direction (sum score) | Vertical trust in both directions (sum score) | Supervisors do not care about workers’ problems | Supervisors take decisions free of personal bias | Supervisors do care about problems or take decisions free of bias  (sum score) | Supervisors do care about problems and take decisions free of bias  (sum score) |
|  | PR (95% CI) | PR (95% CI) | PR (95% CI) | PR (95% CI) | PR (95% CI) | PR (95% CI) | PR (95% CI) | PR (95% CI) |
| Poor SRH | **0.72 (0.59-0.90)** | 0.95 (0.61-1.47) | 1.43 (0.81-2.54) | 0.97 (0.56-1.70) | 1.13 (0.94-1.37) | 0.87 (0.71-1.06) | 0.98 (0.75-1.29) | 0.81 (0.61-1.08) |
| Back pain | 1.01 (0.82-1.24) | 1.18 (0.80-1.73) | 1.20 (0.72-2.00) | 1.19 (0.74-1.93) | 1.13 (0.96-1.32) | 0.99 (0.83-1.20) | **0.77 (0.62-0.96)** | 0.81 (0.65-1.01) |
| Sleeplessness | **0.76 (0.58-0.99)** | 0.99 (0.60-1.61) | 0.92 (0.53-1.62) | 0.76 (0.45-1.27) | 1.08 (0.86-1.35) | 0.83 (0.65-1.06) | **0.73 (0.53-0.99)** | 0.73 (0.54-1.00) |
| Headache | 1.04 (0.89-1.22) | 1.04 (0.80-1.36) | 0.83 (0.61-1.13) | 0.93 (0.71-1.21) | 0.94 (0.83-1.06) | 1.00 (0.81-1.14) | 1.05 (0.86-1.28) | 1.07 (0.88-1.31) |
| Cold | 1.08 (0.87-1.32) | 0.83 (0.64-1.09) | 1.05 (0.68-1.62) | 1.03 (0.69-1.53) | 0.88 (0.75-1.03) | **0.81 (0.69-0.94)** | 0.86 (0.70-1.06) | 0.90 (0.73-1.11) |
| Jaundice | 0.91 (0.73-1.15) | 0.85 (0.61-1.18) | 0.95 (0.61-1.48) | 0.86 (0.57-1.29) | 0.97 (0.81-1.18) | 0.85 (0.69-1.04) | 0.97 (0.72-1.30) | 0.90 (0.67-1.22) |
| Stomach problems | 1.07 (0.75-1.51) | 0.78 (0.49-1.23) | 0.58 (0.32-1.05) | 0.70 (0.43-1.15) | 0.80 (0.61-1.04) | 1.34 (0.97-1.86) | **1.80 (1.01-3.22)** | **2.03 (1.14-3.62)** |
| Muscle cramp | 1.06 (0.82-1.36) | 1.12 (0.73-1.71) | 1.00 (0.58-1.71) | 1.07 (0.66-1.76) | 0.83 (0.68-1.00) | **0.77 (0.63-0.93)** | **0.75 (0.57-0.98)** | 0.86 (0.66-1.12) |
| Eye problems | 0.93 (0.66-1.32) | 1.22 (0.62-2.40) | 1.56 (0.62-3.95) | 1.36 (0.56-3.29) | 1.11 (0.85-1.46) | 0.85 (0.63-1.16) | 1.19 (0.76-1.84) | 0.88 (0.56-1.40) |

*Poisson regression results in form of prevalence ratios (PR) with respective 95% confidence intervals (CI). Adjusted for sex, marital status, education and tobacco use. Significant findings highlighted in bold.

**Supplementary Table: Interaction analysis results for social stressors, social resources and sex among n= 1,118 ready-made garment workers**

|  | Social conflict at work | | | Social support at work | | |
| --- | --- | --- | --- | --- | --- | --- |
|  | Bullied by colleagues *sex | Bullied by supervisor *sex | Bullied by either colleagues or supervisor  (sum score)*sex | Support from colleagues*sex | Support from supervisor*sex | Support from either colleagues or supervisor  (sum score)*sex |
|  | p-value | p-value | p-value | p-value | p-value | p-value |
| Poor SRH | 0.23 | 0.55 | 0.29 | 0.20 | 0.93 | 0.64 |
| Back pain | 0.30 | 0.70 | 0.41 | 0.07 | 0.79 | 0.26 |
| Sleeplessness | 0.51 | 0.62 | 0.53 | 0.32 | 0.12 | 0.19 |
| Headache | **0.04*** | 0.20 | 0.06 | 0.08 | 0.17 | 0.08 |
| Cold | 0.36 | 0.40 | 0.97 | 0.75 | 0.87 | 0.80 |
| Jaundice | 0.13 | 0.28 | 0.75 | 0.11 | 0.69 | 0.28 |
| Stomach problems | 0.22 | 0.64 | 0.34 | 0.86 | 0.54 | 0.93 |
| Muscle cramp | **0.00*** | **0.01*** | **0.00*** | **0.01*** | 0.90 | 0.09 |
| Eye problems | 0.87 | 0.59 | 0.84 | 0.39 | 0.52 | 0.69 |

*Significant values <0.05.

**Supplementary Table: Interaction analysis results for social stressors, social resources and sex among n= 1,118 ready-made garment workers**

|  | Hierarchical interactions at work | | | Leadership at work | | |
| --- | --- | --- | --- | --- | --- | --- |
|  | Trust in information from management*sex | Management trusts the employees*sex | Vertical trust in one direction (sum score)*sex | Supervisors do not care about workers’ problems *sex | Supervisors take decisions free of personal bias *sex | Supervisors do care about problems or take decisions free of bias  (sum score)*sex |
|  | p-value | p-value | p-value | p-value | p-value | p-value |
| Poor SRH | 0.25 | 0.16 | 0.10 | 0.14 | 0.46 | 0.51 |
| Back pain | 0.08 | 0.68 | 0.13 | 0.66 | 0.20 | 0.24 |
| Sleeplessness | 0.16 | 0.26 | 0.11 | 0.90 | 0.85 | 0.79 |
| Headache | 0.84 | 0.48 | 0.58 | 0.11 | 0.44 | 0.44 |
| Cold | 0.69 | **0.03*** | 0.19 | 0.75 | 0.26 | 0.43 |
| Jaundice | 0.80 | 0.96 | 0.91 | 0.67 | 0.09 | 0.64 |
| Stomach problems | 0.92 | 0.55 | 0.84 | 0.90 | 0.81 | 0.95 |
| Muscle cramp | 0.48 | 0.75 | 0.71 | 0.29 | 0.43 | 0.15 |
| Eye problems | 0.24 | 0.39 | 0.14 | 0.96 | 0.84 | 0.96 |

*Significant values <0.05.

**Supplementary Table: Interaction analysis results for social stressors, social resources and dichotomized age among n= 1,118 ready-made garment workers**

|  | Social conflict at work | | | Social support at work | | |
| --- | --- | --- | --- | --- | --- | --- |
|  | Bullied by colleagues*age | Bullied by supervisor*age | Bullied by either colleagues or supervisor  (sum score) *age | Support from colleagues*age | Support from supervisor*age | Support from either colleagues or supervisor  (sum score) *age |
|  | p-value | p-value | p-value | p-value | p-value | p-value |
| Poor SRH | 0.43 | 0.44 | 0.82 | 0.23 | 0.29 | 0.21 |
| Back pain | 0.50 | 0.31 | 0.34 | 0.92 | 0.71 | 0.83 |
| Sleeplessness | 0.10 | 0.33 | 0.11 | 0.10 | **0.04*** | 0.06 |
| Headache | 0.63 | 0.91 | 0.91 | 0.05 | **0.02*** | **0.03*** |
| Cold | 0.29 | 0.53 | 0.36 | 0.61 | 0.49 | 0.70 |
| Jaundice | **0.04*** | **0.04*** | **0.03*** | 0.58 | 0.94 | 0.84 |
| Stomach problems | 0.58 | 0.57 | 0.67 | **0.05*** | **0.04*** | 0.05 |
| Muscle cramp | 0.96 | 0.71 | 0.95 | 0.18 | 0.19 | 0.23 |
| Eye problems | 0.32 | 0.51 | 0.31 | 0.72 | 0.65 | 0.77 |

*Significant values <0.05.

**Supplementary Table: Interaction analysis results for social stressors, social resources and dichotomized age among n= 1,118 ready-made garment workers**

|  | Hierarchical interactions at work | | | Leadership at work | | |
| --- | --- | --- | --- | --- | --- | --- |
|  | Trust in information from management *age | Management trusts the employees*age | Vertical trust in one direction (sum score) *age | Supervisors do not care about workers’ problems*age | Supervisors take decisions free of personal bias *age | Supervisors do care about problems or take decisions free of bias  (sum score) *age |
|  | p-value | p-value | p-value | p-value | p-value | p-value |
| Poor SRH | 0.70 | 0.22 | 0.40 | 0.70 | 0.21 | 0.18 |
| Back pain | 0.50 | 0.52 | 0.49 | 0.73 | 0.94 | 0.73 |
| Sleeplessness | 0.45 | 0.16 | 0.27 | 0.65 | **0.05*** | **0.04*** |
| Headache | 0.06 | 0.11 | 0.07 | 0.91 | 0.07 | **0.05*** |
| Cold | 0.17 | 0.47 | 0.28 | 0.74 | 0.82 | 0.91 |
| Jaundice | 0.45 | 0.19 | 0.27 | 0.10 | 0.17 | 0.42 |
| Stomach problems | **0.01*** | **0.04*** | **0.02*** | 0.52 | **0.01*** | **0.01*** |
| Muscle cramp | 0.06 | **0.04*** | **0.04*** | 0.77 | 0.30 | 0.10 |
| Eye problems | 0.23 | 0.54 | 0.31 | 0.58 | 0.49 | 0.78 |

*Significant values <0.05.
